# Comparison of Humphrey Field Analyzer and imo visual field test results in patients with glaucoma and pseudo-fixation loss

**DOI:** 10.1371/journal.pone.0224711

**Published:** 2019-11-07

**Authors:** Hiroyasu Goukon, Kazunori Hirasawa, Masayuki Kasahara, Kazuhiro Matsumura, Nobuyuki Shoji

**Affiliations:** 1 Graduate School of Medical Science, Kitasato University, Kanagawa, Japan; 2 Department of Ophthalmology, School of Medicine, Kitasato University, Kanagawa, Japan; 3 Moorfields Eye Hospital NHS Foundation Trust and University College London, Institute of ophthalmology, London, United Kingdom; LV Prasad Eye Institute, INDIA

## Abstract

The aim of this cross-sectional study was to evaluate the results of a visual field (VF) test for patients with glaucoma and *pseudo-fixation loss*. These patients exhibit fixation loss (FL) rates >20% with the Humphrey Field Analyzer (HFA); however, actual fixation stabilizes when a head-mounted perimeter (imo) is used. This device is able to adjust the stimulus presentation point by tracking eye movements. We subjected 54 eyes of 54 patients with glaucoma and pseudo-FL to the HFA 30–2 or 24–2 Swedish Interactive Threshold Algorithm -Standard protocol. All patients also underwent the imo 30–2 or 24–2 Ambient Interactive Zipper Estimated Sequential Testing protocol after HFA measurement. We compared HFA and imo reliability indices [including false-positive (FP) responses, false-negative (FN) responses, and FL rate], global indices [including mean deviation (MD), visual field index (VFI), and pattern standard deviation (PSD)], and retinal sensitivity for each test point. There were no significant differences in MD, VFI, and PSD between HFA and imo, and these measures were strongly correlated (r > 0.96, p < 0.01). There were no significant differences in FP and FN between both devices, while FL measured with HFA (27.5%) was significantly reduced when measured with imo (13.2%) (p < 0.01). There was no correlation in FL and FN between both devices, and a weak correlation for FP (r = 0.29, p = 0.04). At each test point, retinal sensitivity averaged 1.7 dB higher with HFA, compared with imo (p < 0.01). There was no significant variability in global indices in patients with pseudo-FL. The FP response rate might have influenced measures of FL in patients with glaucoma and pseudo-FL.

## Introduction

The Humphrey Field Analyzer (HFA: Carl Zeiss Meditec, Dublin, CA, USA) is a standard automated perimetry device currently in worldwide use, used to detect and monitor patients with suspected visual field (VF) damage. Although the reliability of VF results is important when ophthalmologists evaluate glaucoma progression, results can also be influenced by patients’ subjective responses. The reliability of VF results measured with HFA is calculated according to three indices including the fixation loss (FL) rate, false-positive (FP) response rate, and the false-negative (FN) response rate. A reliability criterion for FL is 20%, FP is 15%, and FL is 33%, as determined by the Swedish Interactive Threshold Algorithm-Standard (SITA-Standard) [1].

VF measures retinal sensitivity at a given location while fixing the gaze on a central point. Fixation monitoring during VF measurement is important [2–4]. Previous studies reported that actual eye movements during VF measurement, recorded with an HFA gaze tracking system, were related to the reproducibility of results [5,6], and structure–function relationships in patients with glaucoma [7]. There are a number of reports about the relationships between the VF results and eye movements during VF measurement [4,8–21].

Apart from the actual eye movements that occur during measurement, clinicians occasionally encounter cases of *pseudo-FL*. Here, patients express stable fixation on a monitor; however, the FL rate is >20% even when the Mariotte blind spot is set to the proper location for that patient. Very few reports describe VF in patients with pseudo-FL because the eye movements must be controlled during VF measurement to obtain accurate results. Previous studies reported that, in patients with glaucoma, eye movements within 2 degrees were demonstrated in approximately 70% of the overall fixation time [5,6]. This same phenomenon was additionally observed in well-trained healthy young participants [22].

The imo (CREWT Medical Systems, Tokyo, Japan) is a new, commercially available head-mounted perimeter [23]. The imo can measure VF in nearly all the same positions as HFA. This perimeter allows for real-time automatic gaze tracking by monitoring pupillary movements. With imo, we can also present stimuli in adjusted test points, determined in accordance with eye movements. A previous study found that imo exhibited similar VF sensitivity as the HFA [23]. In theory, imo allows for accurate measurement of VF without the unwanted effects of eye movements during the VF task. Therefore, the aim of this study was to analyze VF measurements obtained using the imo in patients with glaucoma and pseudo-FL.

## Methods

This study followed the tenets of the Declaration of Helsinki, and all participants provided written informed consent after the Institutional Review Board of Kitasato University Hospital (no. B16–101) approved the study.

Fifty-seven eyes of 57 patients with glaucoma, all of whom visited the Kitasato University Hospital glaucoma outpatient clinic between May 2016 and September 2016 were subjected to VF measurements. In addition to glaucoma, all patients were diagnosed with pseudo-FL, as per the latest HFA 24–2 or 30–2 SITA-Standards.

Diagnosis of pseudo-FL was based on observed expression of >20% FL rate, even after rechecking the Mariotte blind spot at least once during measurement. Stable fixation was demonstrated in a monitor with HFA. Before VF measurement, all patients received comprehensive ophthalmic examinations, including noncycloplegic refraction testing, visual acuity testing at 5 meters using a Landolt ring chart, intraocular pressure measurement, and slit-lamp and fundus examination by a glaucoma specialist (MK, KM, or NS). We included patients who were 20–70 years old, with a best-corrected decimal visual acuity of 0.8 (logarithm of the minimal angle of resolution [LogMAR] 0.1) or better, spherical power within ±6 diopter and cylindrical power < 1.5 diopter and FP measured with HFA was <15%. We excluded patients with tracking failure on imo.

### Head-mounted perimeter “imo”

In brief, the head-mounted perimeter imo consists of the main perimeter unit, a user control tablet, and a patient response button [23]. The imo is equipped with two separate sets of optical systems and pupil-monitoring systems for the right and left eyes. Therefore, the imo can independently present targets, or carry out pupil monitoring, for either eye. Using a full high-definition transmissive light emitting diode and high-intensity light emitting diode backlights, imo can display a test target under the same test conditions as the HFA. The imo has an automatic eye-tracking system that follows eye movement from the fixation point and corrects the location of the target presentation. The test pattern used for imo is compatible with the 24–2 and 30–2 test pattern in HFA. imo uses the AIZE (Ambient Interactive Zipper Estimated Sequential Testing) measurement strategy during modified Zipper Estimated Sequential Testing. With AIZE, which is an original imo measurement strategy, thresholds are determined using Bayesian inference and the maximum likelihood method. The system triggers one reply in the periphery and displays a simulated object while the patient interacts with neighboring checkpoints in order to determine retina sensitivity thresholds. Other detailed features of the imo are described in a previous report [23]. The measurement conditions for imo and HFA that were used in the current study are shown in **Table 1**.

**Table 1:** Measurement conditions for imo and the Humphrey Field Analyzer (HFA).

parameter	imo	HFA
Background luminance	31.5 apostilb	31.5 apostilb
Maximum stimulus intensity	10,000 apostilb	10,000 apostilb
Stimulus presentation time	0.2 seconds	0.2 seconds
Stimulus size	Goldmann III	Goldmann III
Test point program	24–2 or 30–2	24–2 or 30–2
Test strategy	AIZE	SITA-Standard
Fixation environment	monocular measurement under the both eyes opening	monocular measurement under covering fellow eye
Fixation monitoring	Automatic tracking system Heijl–Krakau method	Heijl–Krakau method Gaze-Tracking method

AIZE, Ambient Interactive Zipper Estimated Sequential Testing. SITA, Swedish Interactive Threshold Algorithm.

HFA and imo were performed on the same day, with all patients undergoing imo after a break. Reliability indices including FL, FP, and FN rate and global indices including mean deviation (MD), visual field index (VFI), and pattern standard deviation (PSD) were compared between HFA and imo. Differences in retinal sensitivity at each test point were also calculated.

### Statistical analysis

All statistical analyses were performed using the statistical software packages SPSS version 22.0 (IBM Japan, Ltd., Tokyo, Japan). Wilcoxon signed-rank tests were used for between-group comparisons. Pearson product–moment correlation coefficients and Bland–Altman analyses were used to compare HFA parameters with the corresponding imo parameters.

## Results

Out of 57 eyes, we excluded 3 eyes because of tracking failure on imo. Finally, 54 eyes of 54 patients with glaucoma were analyzed. Participants’ demographics and ocular characteristics are summarized in **Table 2**.

**Table 2:** Participants’ demographic and ocular characteristics.

Parameter	Mean ± standard deviation (range)
Eyes (right/left)	54 (24/30)
Sex (male/female)	34/20
Age (years)	62.4 ± 9.9 (40 to 70)
Spherical refraction (D)	−0.70 ± 2.19 (−6.00 to 2.75)
Cylinder refraction (D)	−0.53 ± 0.63 (−1.50 to 0.00)
Visual Acuity (logMAR)	−0.04 ± 0.06 (−0.08 to 0.10)
Intra-ocular pressure (mmHg)	13.7 ± 4.2 (7.0 to 22.0)
HFA mean deviation (dB)	−5.8 ± 6.9 (−27.7 to 2.0)
HFA visual field index (%)	82.7 ± 21.2 (15 to 100)
HFA pattern standard deviation (dB)	6.6 ± 4.7 (1.2 to 15.9)
HFA fixation loss rate (%)	27.5 ± 7.08 (20 to 56)
Type of glaucoma (eyes)	
Primary open-angle glaucoma	23
Normal-tension glaucoma	18
Secondary glaucoma	8
Pre-perimetric glaucoma	3
Primary closed-angle glaucoma	2

LogMAR: logarithm of the minimal angle of resolution.

**Fig 1:**
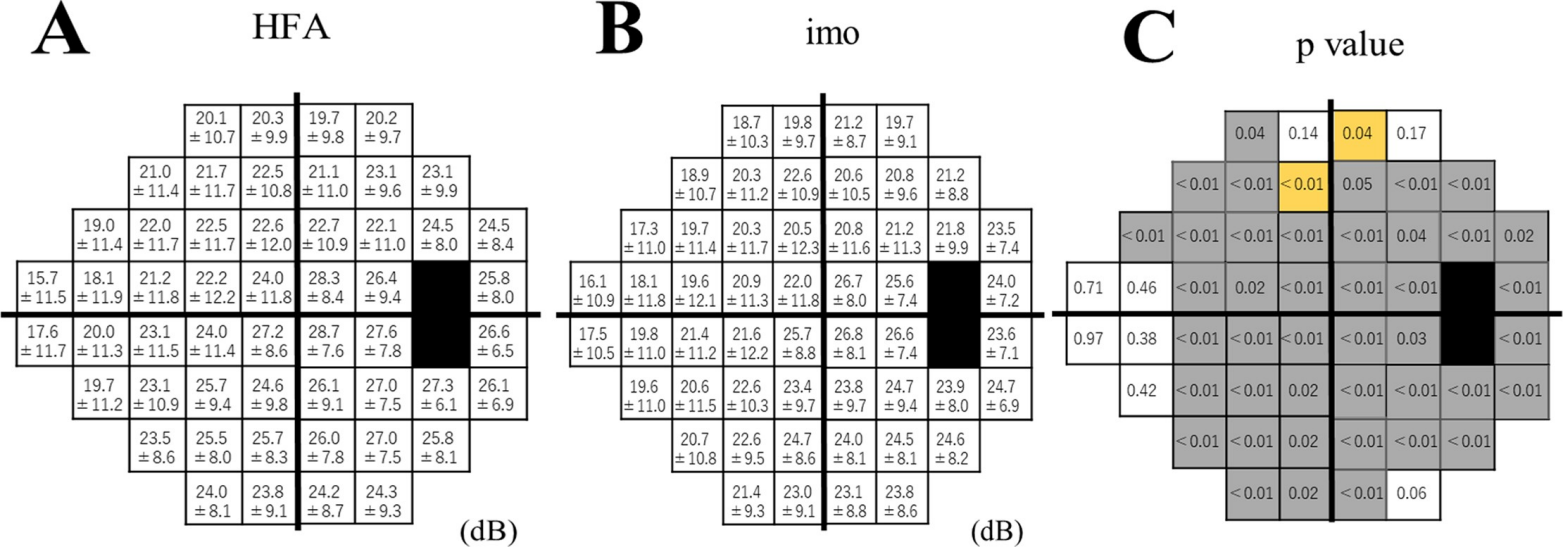
Comparison of the actual threshold value at each test point between the Humphrey Field Analyzer (HFA) and imo. The data are expressed as mean ± standard deviation. The actual threshold value at each test point between HFA (**Fig 1A**) and imo (**Fig 1B**), with corresponding p values (**Fig 1C**), are shown. The black area represents the blind spot. Data are expressed in dB. The grey area represents high HFA thresholds, compared with imo. The orange area represents high imo thresholds, compared with HFA.

**Fig 1** shows retinal sensitivity measures, as determined by HFA and imo. Retinal sensitivity at each HFA test point was significantly lower than values obtained with imo at 2 points. The retinal sensitivities at each test point averaged 1–2 dB higher with HFA than with imo.

**Table 3** presents comparisons of reliability and global indices between HFA and imo. For the reliability indices, there was a significant difference in the FL rate between HFA (27.5%) and imo (13.2%) (p < 0.01); however, there was no significant difference in FP and FN.

**Table 3:** Comparison of reliability indices and global indices measured with the Humphrey Field Analyzer (HFA) and imo.

Parameter	HFA	imo	p value
Reliability indices			
Fixation loss rate (%)	27.5 ± 7.1 (20 to 56)	13.2 ± 17.3 (0 to 57)	<0.001
False-positive rate (%)	4.3 ± 4.0 (0 to 15)	5.6 ± 9.0 (0 to 38)	0.74
False-negative rate (%)	4.1 ± 5.4 (0 to 21)	6.2 ± 9.4 (0 to 36)	0.18
Global indices			
Mean deviation (dB)	−5.8 ± 6.9 (−27.7 to 2.0)	−5.5 ± 6.8 (−24.0 to 1.5)	0.12
Visual field index (%)	82.7 ± 21.2 (15 to 100)	82.9 ± 22.3 (14 to 100)	0.55
Pattern standard deviation (dB)	6.6 ± 4.7 (1.2 to 15.9)	6.5 ± 4.4 (1.0 to 14.7)	0.99

Data are expressed as mean ± standard deviation (minimum to maxim). HFA: Humphrey Field Analyzer

**Fig 2:**
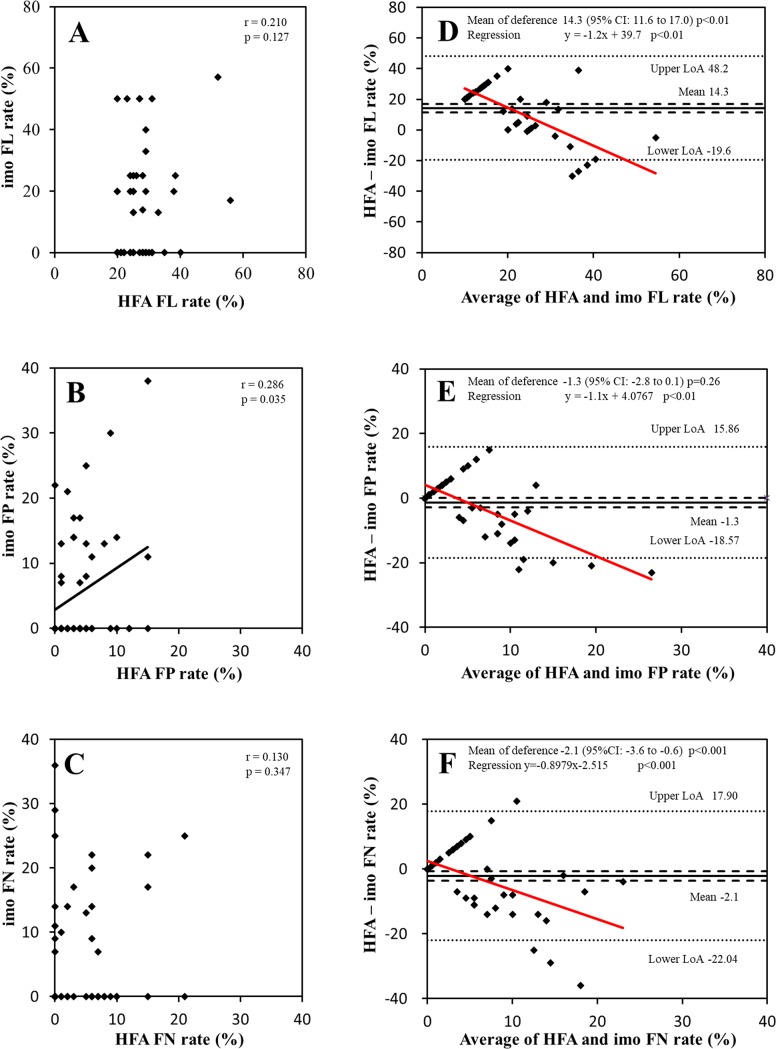
Scatter plots and Bland–Altman plots of the reliability indices between the Humphrey Field Analyzer (HFA) and imo. The scatter and Bland–Altman plots for fixation loss (FL, **A and D**), false-positive (FP, **B and E**), and false-negative (FN, **C and F**) rate are shown in these figures. Correlation coefficient (r) and p values are depicted in the scatter plots (**A–C**). The mean difference (black solid line) and its 95% confidence interval (CI, dashed line) and limits of agreement (LoA, dotted line) are shown in the Bland–Altman plots (**D–F**). The solid red line represents the best-fit line for proportional bias.

**Fig 2** shows the scatter plot and Bland–Altman analysis of reliability indices measured with HFA and imo. A positive weak correlation in FP was found between HFA and imo (r = 0.29, p = 0.04), while there was no significant correlation in FL and FN (**Fig 2A–2C**). For the Bland–Altman analysis, FL and FN demonstrated a fixed bias (p < 0.01), and a proportional bias was demonstrated for all reliability indices (p < 0.01, **Fig 2D–2F**).

**Fig 3:**
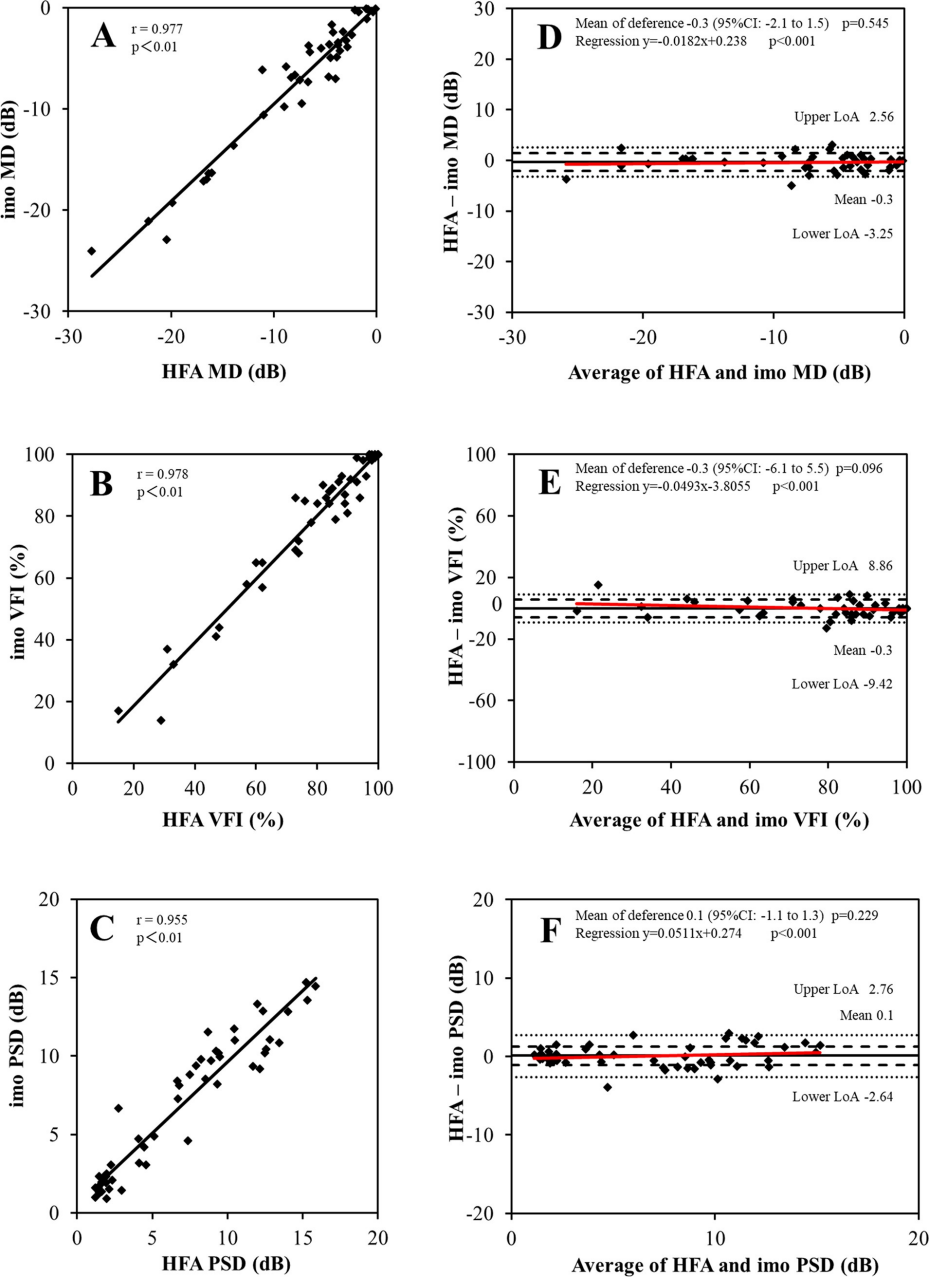
Scatter plots and Bland–Altman plots of the global indices between the Humphrey Field Analyzer (HFA) and imo. The scatter plots and Bland–Altman plots of mean deviation (MD, **A and D**), visual field index (VFI, **B and E**), and pattern standard deviation (PSD, **C and F**) rate are shown in these figures, respectively. Correlation coefficient (r) and p values are shown in the scatter plots (**A–C**). The mean difference (black solid line) and its 95% confidence interval (CI, dashed line) and limits of agreement (LoA, dotted line) are shown in Bland–Altman plots (**D–F**). The solid red line shows the best-fit line for proportional bias.

For the global indices, there were no significant differences between HFA and imo for MD, VFI, and PSD (**Table 3**). **Fig 3** shows the result of the scatter plot and Bland–Altman analysis in global indices measured with HFA and imo. Strong correlation was demonstrated in MD (r = 0 .98, p < 0.01), VFI (r = 0.98, p < 0.01), and PSD (r = 0.96, p < 0.01) between HFA and imo (**Fig 3A–3C**). For the Bland–Altman analysis, there was no fixed bias between HFA and imo in any global index; whereas, proportional bias was demonstrated for each global index (p < 0.01, **Fig 3D–3F**).

**Fig 4:**
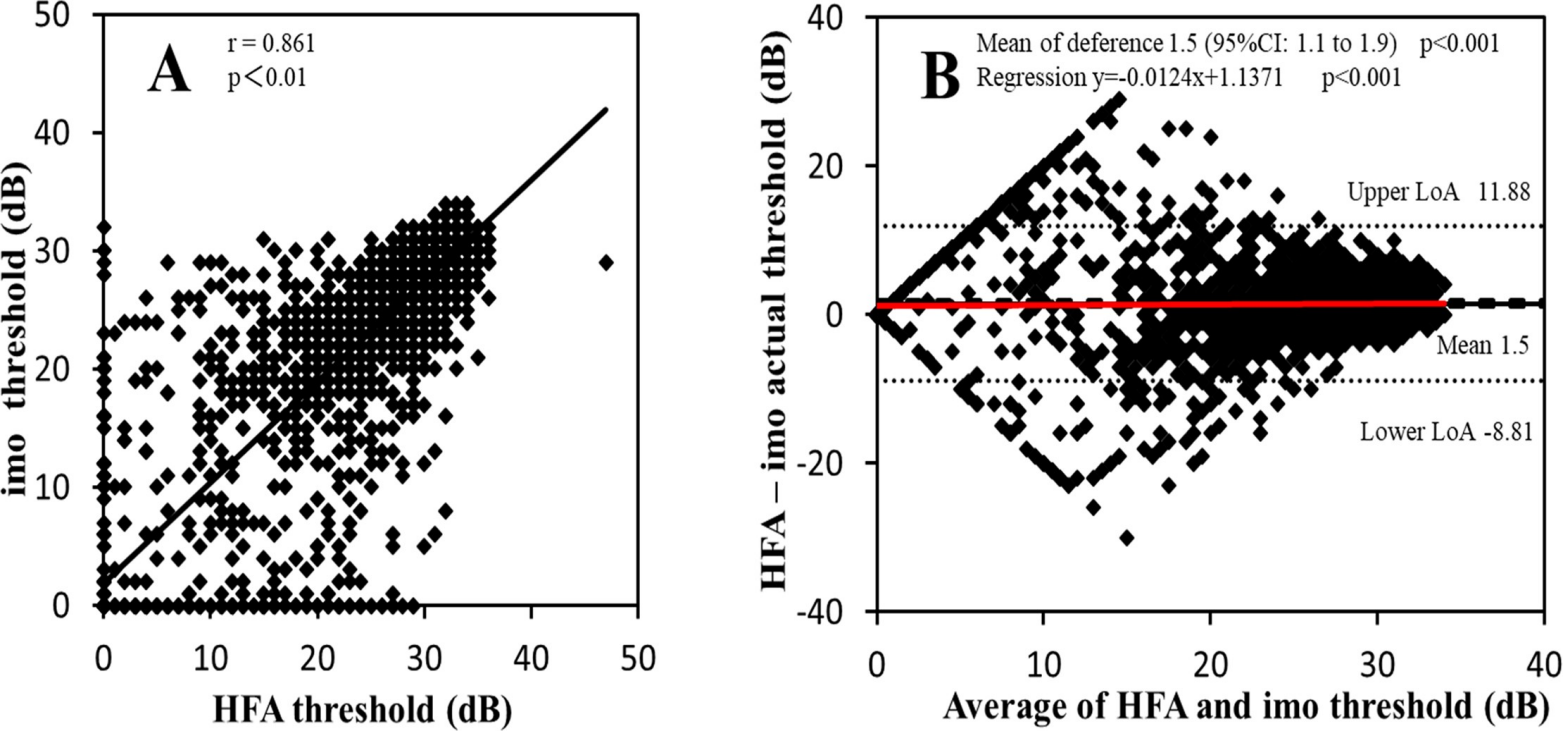
Scatter plots and Bland–Altman plots of visual field (VF) sensitivity at each test point between the Humphrey Field Analyzer (HFA) and imo. The scatter plots and Bland–Altman plots of visual field (VF) sensitivity at each test point (**A and B**) are shown in these figures, respectively. Correlation coefficient (r) and p values are shown in the scatter plots (**A**). The mean difference (black solid line) and its 95% confidence interval (CI, dashed line) and limits of agreement (LoA, dotted line) are shown in the Bland–Altman plots (**B**). The solid red line shows the best-fit line for proportional bias.

**Fig 4** shows the result of scatter plot and Bland–Altman analysis of VF sensitivity at each test point, as measured with HFA and imo. Strong correlation was demonstrated between HFA and imo (r = 0.86, p < 0.01) (**Fig 4A**). For the Bland-Altman analysis, both fixed and proportional biases were also demonstrated in VF sensitivity at each test point between HFA and imo (p < 0.01, **Fig 4B**).

## Discussion

We evaluated VF in patients with glaucoma and pseudo-FL using imo. We found that both the global indices (MD, VFI, and PSD) and retinal sensitivities were strongly correlated for HFA and imo at each test point. There were no significant differences in global indices between either parameter, while retinal sensitivity at each test point averaged 1.7 dB higher for HFA, compared with imo. There were large-scale agreements in reliability indices (FL, FP, and FN) between HFA and imo, and there was a significant difference in FL between the two perimeters. A positive weak correlation was found for FP.

FL measured with HFA (27.5%) was significantly reduced when measured by imo (13.2%). FL rates are calculated using the Heijl–Krakau method for both devices. The frequency of stimulus presentation on the Mariotte blind spot was set to 5% of the number of total stimulus presentations [1]. However, the number of total stimulus presentations could differ between the two devices because different test strategies were used for HFA (SITA-Standard) and imo (AIZE). With imo, when slight eye movements occurred during VF measurement, the device compensated for stimulus presentation on the Mariotte blind spot using an automatic tracking system. During HFA, the participant can hear the motor sound when each stimulus is presented. In contrast, there are no sounds during imo measurement. These reasons may explain why FL was decreased for imo.

The positive weak correlation was found for FP. A previous study reported that FP affected test–retest variability at each test point or MD [6]. FP is calculated in HFA as the ratio of the number of responses within 180 to 200 msec against each stimulus presentation [24]. This cut-off value is based on the average response time against the stimulus presentation [25]. In contrast, for imo, FP is calculated as the ratio of the number of responses against the stimulus, which is 9 dB dimmer than the threshold of the test location (already defined) (imo instruction manual). A high FP rate means that the number of responses is large even though they were not actually seen. If a patient kept looking at the fixation point during the test, he or she should not respond during stimulus presentation on the Mariotte blind spot. However, the rate of FL could be high even though the participant kept looking at the fixation target. A previous study about the effects of induced FP during testing on VF found that as the induced FP rate increased, so too did FL [26]. Given that all patients in this study demonstrated pseudo-FL, the FP responses might have been affected.

Global indices and retinal sensitivity measures were strongly correlated between HFA and imo at each test point. However, retinal sensitivity at each test point was averaged 1.7 dB higher with HFA than with imo. Matsumoto et al. reported that mean sensitivity was highly correlated between HFA and imo (r = 0.94–0.96) [23]. In the current study, the MD demonstrated similar findings (r = 0.98). Matsumoto et al. also revealed that there was no significant difference in the mean sensitivity value between HFA and imo. This contradiction may have occurred because of the order of examination. In this study, each patient underwent imo measures *after* HFA. Kelly et al. reported that the sensitivity decreased significantly by 0.13 dB at the second testing secondary to fatigue [27]. Although we were careful to provide sufficient breaks to the patients between the two measurement sessions, fatigue might have affected the sensitivity measures at each test point. Additionally, the possibility of a learning effect cannot be excluded. All participants received imo for the first time, but we performed a quick demonstration before taking actual measurements. A previous study about learning effects during automated static perimetry found that sensitivity improved with the second measurement by approximately 0.3–5.0 dB in patients with glaucoma [28] and 0.6–1.3 dB in healthy participants [29]. The difference of approximately 1.7 dB between HFA and imo measures may have been influenced by fatigue and/or learning effects.

The current study had some limitations. Our study cohort was rather small. Fixation on the monitor was stable; however, it is possible that the line of sight deviated from time to time. Measurements taken by imo were always performed after HFA, patients had no prior exposure to imo, and we cannot rule out a learning effect. Finally, imo having been monocular measurement under the both eyes opening and the effect of pupil diameter due to shielding.

In conclusion, global indices did not significantly vary between HFA and imo and there were strong correlations in these parameters in patients with glaucoma and pseudo-FL. However, reliability index variability was large between the HFA and imo. FP response might have influenced the high FL responses in patients with glaucoma and pseudo-FL.

## Supporting information

S1 File Supplementary data of this study. https://figshare.com/s/405386bebbc2a44f7517.
(XLSX)
